# Huntingtin *HTT1a* is generated in a CAG repeat-length-dependent manner in human tissues

**DOI:** 10.1186/s10020-024-00801-2

**Published:** 2024-03-08

**Authors:** Franziska Hoschek, Julia Natan, Maximilian Wagner, Kirupa Sathasivam, Alshaimaa Abdelmoez, Björn von Einem, Gillian P. Bates, G. Bernhard Landwehrmeyer, Andreas Neueder

**Affiliations:** 1 Department of Neurology, University Hospital Ulm, 89081 Ulm, Germany; 2 Huntington’s Disease Centre, Department of Neurodegenerative Disease, Queen Square Institute of Neurology, University College London, WC1N 3BG London, UK; 3 Department of Pharmaceutical Organic Chemistry, Faculty of Pharmacy, Assiut University, Assiut, Egypt

**Keywords:** Huntington disease, Digital PCR, Neurodegeneration, Alternative splicing, Biomarker

## Abstract

**Background:**

The disease-causing mutation in Huntington disease (HD) is a CAG trinucleotide expansion in the huntingtin (*HTT*) gene. The mutated CAG tract results in the production of a small RNA, *HTT1a*, coding for only exon 1 of HTT. *HTT1a* is generated by a block in the splicing reaction of *HTT* exon 1 to exon 2 followed by cleavage in intron 1 and polyadenylation. Translation of *HTT1a* leads to the expression of the highly toxic HTT exon 1 protein fragment. We have previously shown that the levels of *HTT1a* expression in mouse models of HD is dependent on the CAG repeat length. However, these data are lacking for human tissues.

**Methods:**

To answer this question, we developed highly sensitive digital PCR assays to determine *HTT1a* levels in human samples. These assays allow the absolute quantification of transcript numbers and thus also facilitate the comparison of *HTT1a* levels between tissues, cell types and across different studies. Furthermore, we measured CAG repeat sizes for every sample used in the study. Finally, we analysed our data with ANOVA and linear modelling to determine the correlation of *HTT1a* expression levels with CAG repeat sizes.

**Results:**

In summary, we show that *HTT1a* is indeed expressed in a CAG repeat-length-dependent manner in human *post mortem* brain tissues as well as in several peripheral cell types. In particular, PBMCs show a statistically significant positive correlation of *HTT1a* expression with CAG repeat length, and elevated *HTT1a* expression levels even in the adult-onset CAG repeat range.

**Conclusions:**

Our results show that *HTT1a* expression occurs throughout a wide range of tissues and likely with all CAG lengths. Our data from peripheral sample sources demonstrate that HTT1a is indeed generated throughout the body in a CAG repeat-length-dependent manner. Therefore, the levels of *HTT1a* might be a sensitive marker of disease state and/or progression and should be monitored over time, especially in clinical trials targeting HTT expression.

**Supplementary Information:**

The online version contains supplementary material available at 10.1186/s10020-024-00801-2.

## Introduction

Expansions of microsatellites, repetitive nucleotide sequences scattered throughout the genome, are the cause of a number of diseases (Neueder 2019). Huntington disease (HD) is the most common disease of a family of disorders that is caused by expansions of a CAG triplet sequence in their coding region (Neueder 2019). Huntingtin (*HTT*), the gene harbouring the expanded CAG repeat in HD, is a ubiquitously expressed, large (67 exons) and alternatively spliced gene that gives rise to several canonical and numerous disease related isoforms (Neueder and Bates 2018). The unstable CAG repeat is located in exon 1 of *HTT* (The Huntington’s Disease Collaborative Research Group 1993) and a CAG tract size of 40 or more invariably leads to HD (Bates et al. 2015). Disease onset, severity and progression are linked to the CAG tract length, with a longer CAG tract leading to onset at a younger age, a more rapid progression, and a shorter life expectancy (Bates et al. 2015). In addition, the CAG tract expands somatically and can reach extreme lengths of hundreds of CAG triplet repeats resulting in *HTT* alleles that reflect the more severe disease form of juvenile-onset HD caused by CAG sizes of approximately 65 and above (Monckton 2021).

Alternative splicing of the *HTT* gene and proteolytic cleavage of the HTT protein generate C-terminally truncated fragments of HTT (Sathasivam et al. 2013; Neueder et al. 2017; Bent et al. 2022). The severity of disease and molecular phenotypes is strongly correlated with smaller N-terminal fragments (Bent et al. 2022). The most toxic fragment of HTT is encoded by exon 1, which includes the expanded CAG tract, and induces both protein and RNA based toxicity (Heinz et al. 2021). We have shown that this fragment is translated from a novel, disease-related isoform of *HTT*, *HTT1a*, that is generated by a block in the splicing of exon 1 to exon 2 (Sathasivam et al. 2013). Consequently, the *HTT1a* mRNA consists of exon 1 and parts of intron 1. *HTT1a* is polyadenylated, exported from the nucleus and translated into the HTT exon1 protein (Sathasivam et al. 2013). Moreover, splicing factors like SRSF6, RNA polymerase II function, *HTT1a* retention in nuclear clusters and other genomically encoded regulatory elements influence the amount of *HTT1a* expression (Sathasivam et al. 2013). Furthermore, we could show that *HTT1a* is generated in *post mortem* brain tissues from HD mutation carriers (Neueder et al. 2017).

Our analysis showed that the production of *HTT1a* is positively correlated with increasing CAG repeat lengths in all knockin mouse models (Sathasivam et al. 2013, Gipson et al. 2013). However, it is still unclear whether this is also the case in human tissue. To answer this question, we developed digital PCR assays that allow absolute quantification of *HTT1a* transcript numbers and exhibit greatly increased sensitivity compared to the previously used qPCR assays. We analysed *post mortem* brain tissue and could confirm our previous findings. Additionally, we analysed peripheral samples: primary fibroblast and lymphoblastoid cell lines, peripheral blood mononuclear cells (PBMCs) and skeletal muscle. We found that lymphoblastoid cells were not suitable for analysis of *HTT1a* expression, because the CAG tract becomes unintentionally unstable regardless of the initial CAG length, likely due to the immortalization procedure. In summary, *HTT1a* expression in all other cell types was strongly correlated to the respective CAG repeat lengths. The peripheral cell types that were analysed are much more easily accessible than brain tissue and can be collected, for example, in the context of clinical trials. In particular, clinical interventions aimed at reducing *HTT* expression or targeting CAG repeat instability will greatly benefit from our work (Tabrizi et al. 2022), as the reduction of *HTT1a* expression is most likely necessary for maximal therapeutic effect.

## Results

### A multiplexed digital PCR assay to analyse HTT1a expression levels in human samples

Digital PCR, in contrast to quantitative PCR (qPCR), is an endpoint PCR method. The basic principle of digital PCR is the random separation of the sample into many individual reactions, where single, parallel PCR reactions take place. These partitions can contain either zero, one, or more than one target molecules (Quan et al. 2018). The primary difference between the available systems is in the separation of the individual reactions. For this mainly droplet/emulsion based, or chip/micro-well based systems are used. We opted to use the chip-based QuantStudio™ 3D Digital PCR System (ThermoFisher). However, we believe that the assays that we have developed could be easily transferred to any platform and technology. After the PCR, positive and negative signals/wells are counted, and quantification of the target sequences is subsequently achieved by using Poisson Plus statistics to calculate the final transcript numbers. For our use, the biggest advantages of digital PCR over qPCR are the greatly increased sensitivity and the fact that no internal or external standards are needed for absolute quantification.

**Fig. 1:**
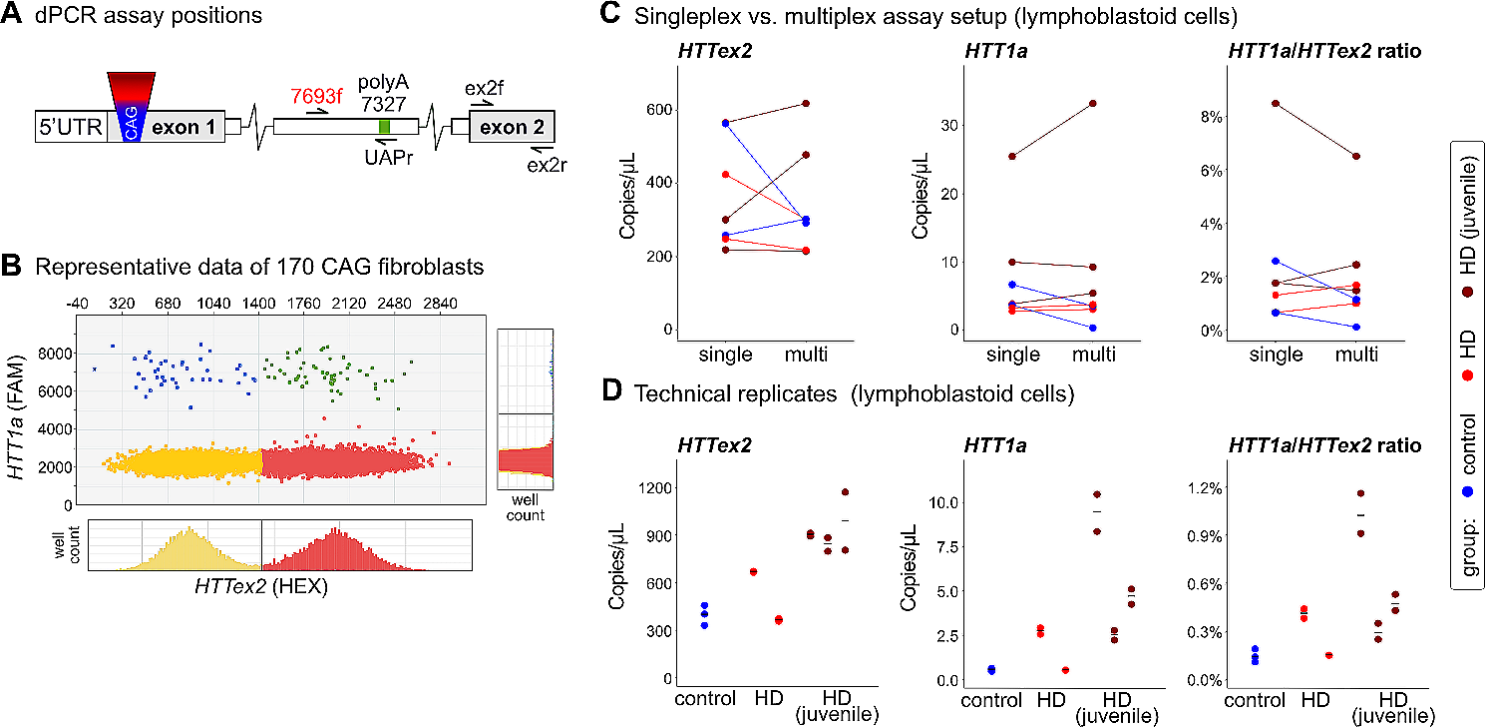
A multiplexed digital PCR assay to analyse HTT1a expression levels in human samples **A** Schematic overview of assay positions within the 5’ region of the *HTT* gene. The cryptic polyA site is 7,327 bp into intron 1. Sequences are listed in Table 1. **B** Exemplary results of a chip-based, multiplexed digital PCR assay. *HTT* exon 2 transcripts (*HTTex2*) are shown on the x-axis and *HTT1a* transcripts on the y-axis. Wells with a positive signal for each assay are shifted towards larger values. Wells that did not contain a target transcript are shown in yellow. Wells that were only positive for either *HTTex2* or *HTT1a* are shown in red or blue, respectively. Wells that contained both target transcripts are shown in green. **C and D** Samples had CAG sizes in the control (control), adult onset (HD) or juvenile-onset range (HD juvenile). **C** Comparison of the performance of individually run assays versus multiplexed assays in primary human lymphoblastoid cell lines. The same cDNAs were used for the comparisons. **D** Assessment of technical inter-chip variation. The same cDNA from human lymphoblastoid cell lines was run on multiple chips. The black line denotes the mean of the individual chips. Each stack represents a different cell line. Ratios were built by dividing the signal of *HTT1a*/*HTT exon 2* for each chip and subsequently calculating the mean for each line.

**Fig. 2:**
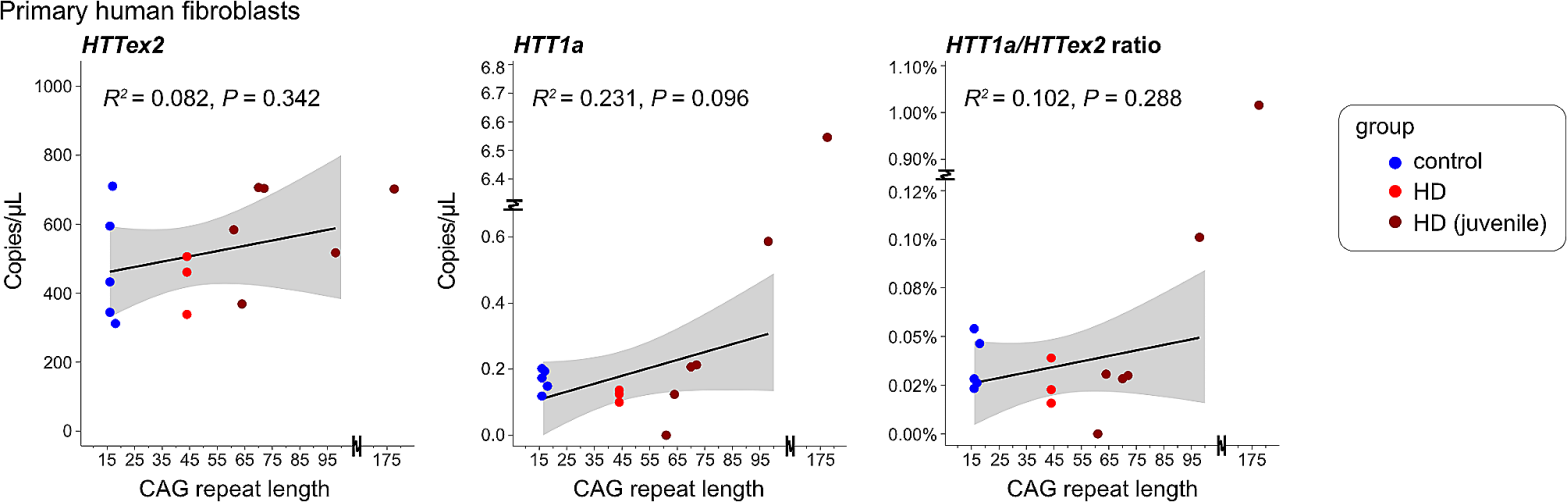
*HTT1a* expression in primary human fibroblasts can only be detected in lines with large CAG expansions *HTT1a* and *HTT* exon 2 (*HTTex2*) expression in primary human fibroblast lines was analysed in lines with a CAG repeat in the control (control), adult onset (HD) or juvenile-onset range (HD juvenile). We could only detect *HTT1a* reliably in the lines containing very long CAG repeats (> 100). The black line represents the linear model of expression level with CAG repeat length calculated from the mean of 3 different time points in culture (see also Figure S1A). R^2^ (coefficient of determination) and *P* value for the fit of the linear modelling are shown. Grey areas represent the standard error of the regression model. We did not include the line with the very large expansion in the modelling to avoid spurious fits.

To establish our assays, we used a primary human fibroblast line with extremely long repeat of over 170 CAGs (GM09197, Coriell), which we also previously analysed by qPCR (Neueder et al. 2017). This very long CAG repeat length led to substantial *HTT1a* expression levels that were easily detectable (Figs. 1 and 2; approx. 1% of *HTT1a* vs total *HTT* expression levels). A schematic overview of the position of the assays within the 5’ region of the *HTT* gene is shown in Fig. 1A. Assay details and sequences are given in Table 1. Figure 1B shows a typical result from a digital PCR chip of the GM09197 fibroblast line. *HTT* exon 2 transcripts (*HTTex2*) are shown on the x-axis and *HTT1a* transcripts on the y-axis. Wells with a positive signal for each assay are shifted towards larger values. Wells that did not contain a target transcript are shown in yellow. Wells that were only positive for either *HTTex2* or *HTT1a* are shown in red or blue, respectively. Wells that contained both target transcripts are shown in green. Histograms for each axis show the thresholds between negative and positive signals (black lines).

**Table 1:** Assay sequences

Assay	Name	Sequence (5’-3’)	Modification
RT-reaction	UAPdT18	GGCCACGCGTCGACTAGTACTTTTTTTTTTTTTTTTTT	
*HTT* exon 2	HTTex2_f	AAAGAAAGAACTTTCAGCTACCAAGAA	
	HTTex2_r	CTGACAGACTGTGCCACTATG	
	HTTex2_p	TCACATATTGTCAGACAATGATTCACACGGTCT	5’-HEX, 3’-BHQ2
*HTT1a*	HTT1a_f	GGATCCACACTCAAAACATTTA	
	UAP18qPCR	CACGCGTCGACTAGTAC	
	HTT1a_p	TCTTATTCAGACAACAAGGAGGAAAAATAAAATACC	5’-FAM, 3’-BHQ1

Nucleotide sequences of the digital PCR assays. Oligonucleotides were ordered from Eurofins genomics. BHQ = Black Hole Quencher; FAM = 6-carboxyfluorescein; HEX = hexachlorofluoresceine

We included *HTT* exon 2 containing transcripts in our analysis to be able to draw conclusions about the expression levels of ‘full-length’ *HTT* mRNA, which has been reported to be downregulated with larger repeat lengths in certain tissues (Neueder et al. 2017; Evers et al. 2015). Naturally, other regions of the *HTT* gene would be feasible to analyse *HTT* full-length expression levels, too. However, we noticed that the best correlation between assays is reached when the assays target sequences in close spatial proximity (data not shown). This is either an effect of reverse transcription from the polyA tail, or alternative splicing, or other RNA modifying events, that potentially modulate the expression of the more distally different isoforms. Our digital PCR system allows multiplexing of two assays. Therefore, we tested if the *HTT1a* and *HTT* exon 2 assays were affected by the multiplexing strategy but could not detect major differences in lymphoblastoid cell lines (Fig. 1C). In particular, the ratio of the two assays was very stable (Fig. 1C, *HTT1a*/*HTTex2* ratio). Regarding the relatively high levels of *HTT1a* in lymphoblastoid lines from control individuals (Fig. 1C), the reader is referred to the text below.

Furthermore, we analysed the inter-chip variation. To this end, the same cDNAs were run on two individual chips and results were compared (Fig. 1D). Although the inter-chip variation was relatively low, we decided to use two chips as a technical replicate for each sample in all following experiments.

### Validation of the multiplexed digital PCR assay

To validate our multiplexed digital PCR assay, we analysed human *post mortem* brain tissues and primary human fibroblast lines, both of which we had analysed previously by qPCR (Neueder et al. 2017). When we analysed fibroblast lines, we were able to robustly detect *HTT* exon 2 in lines with CAG lengths in the control and adult-onset range, however elevated levels of *HTT1a* were only detectable in lines with very long CAG repeats (> approx. 100) (Fig. 2). No statistically significant differences for the group-wise comparisons were detected (Fig. 2). This is in line of what we had observed previously using qPCR assays (Neueder et al. 2017).

**Fig. 3:**
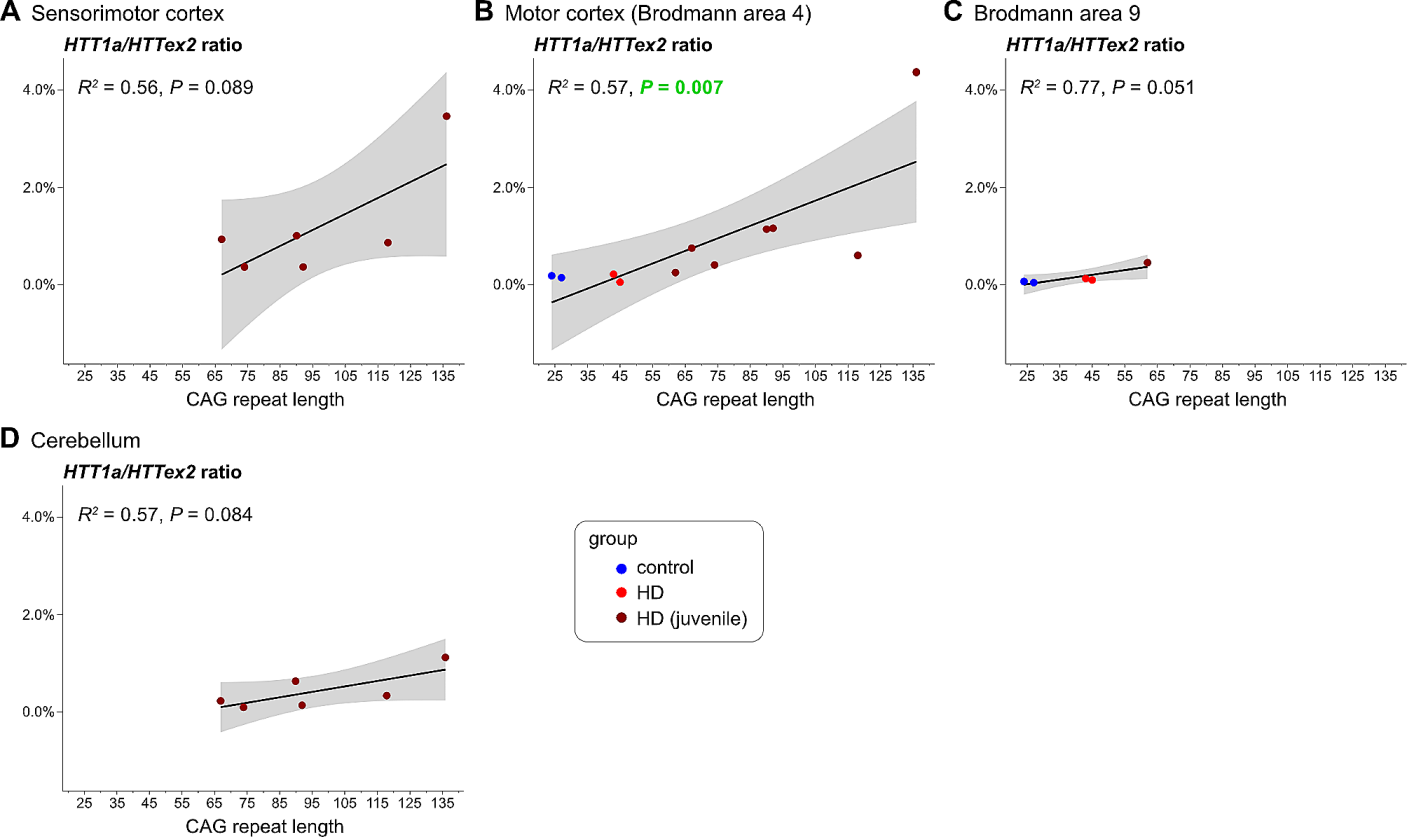
*HTT1a* is generated in a CAG repeat-length-dependent manner in *post mortem* brain regions *HTT1a* normalised to *HTT* exon 2 (*HTTex2*) expression levels in sensorimotor cortex (**A**), motor cortex (BA4) (**B**), BA9 cortex (**C**) and cerebellum (**D**) were analysed from *HTT* mutation carriers with adult-onset CAG repeat lengths (HD) or juvenile-onset CAG repeat lengths (HD juvenile) and control individuals (control), respectively. The black line represents the linear model of expression level with CAG repeat length. R^2^ (coefficient of determination) and *P* value for the fit of the linear modelling are shown. Grey areas represent the standard error of the regression model. For data showing the individual *HTT* isoform expression levels see supplementary Figure S2.

Next, we applied digital PCR to different *post mortem* brain regions (Fig. 3), some of which were analysed previously by qPCR (Neueder et al. 2017) (Fig. 3A and B (juvenile-onset range; CAG > 65) and 3D). In addition, we included samples from *post mortem* BA9 cortex (Fig. 3C). Throughout all brain regions we detected robust normalised *HTT1a* (to *HTTex2*) expression levels in samples with CAG sizes in the juvenile-onset range. Unfortunately, we only had access to two samples with repeats in the adult-onset range and could only analyse 2 out of the 4 brain regions. Normalised *HTT1a* levels in these samples were on par with *HTT1a* levels in control individuals (Fig. 3B and C). Our analysis of motor cortex, for which we also had the largest samples size with CAG repeat lengths up to approximately 140, showed a statistically significant, positively correlated relationship of normalised *HTT1a* levels with CAG repeat length (Fig. 3B). In conclusion, our assays confirmed the previously published data (Neueder et al. 2017) and demonstrated that *HTT1a* is produced in HD patient brains throughout different regions.

### HTT1a expression in lymphoblastoid cell lines might be biased by unintentionally unstable CAG expansions

**Fig. 4:**
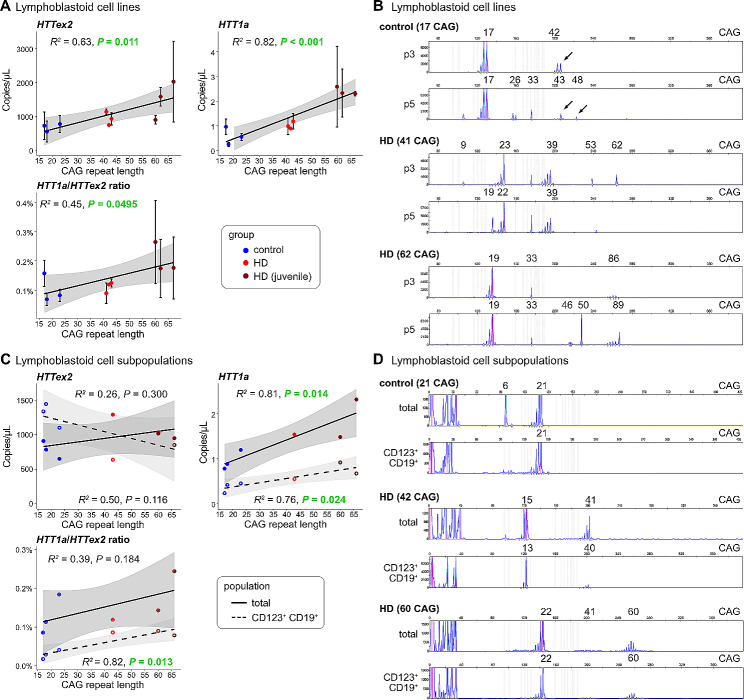
*HTT1a* is generated in a CAG repeat-length-dependent manner in lymphoblastoid cell lines **A***HTT1a* and *HTT* exon 2 (*HTTex2*) expression in human lymphoblastoid cell lines was analysed in lines with a CAG repeat in the control (control), adult-onset (HD) or juvenile-onset range (HD juvenile). The black line represents the linear model of expression level with CAG repeat length calculated from the mean of three different time points in culture ± SEM (see also Figure S1B). R^2^ (coefficient of determination) and *P* value for the fit of the linear modelling are shown. Grey areas represent the standard error of the regression model. **B** Exemplary CAG repeat size traces from lymphoblastoid lines analysed in A. CAG repeat length of the longer allele determined from total blood DNA is given in brackets. **C***HTT1a* and *HTT* exon 2 expression analysis of CD123^+^/CD19^+^ FACS sorted cells (dotted lines) versus the total population (solid lines). Linear modelling was as in A. **D** Exemplary CAG repeat size traces from lymphoblastoid lines analysed in C. CAG repeat length of the longer allele determined from total blood DNA is given in brackets.

Lymphoblastoid cells lines expand almost indefinitely and thus are widely used as a renewable source of patient specimens. Lymphoblastoid lines were purchased from the European Huntington Disease Network (EHDN, www.ehdn.org). Lines were generated by BioRep (Italy, www.biorep.it) by immortalizing B-cells with Epstein Barr virus (EB virus). We analysed lines with CAG repeats in the control (control), adult-onset (HD) or juvenile-onset range (HD juvenile) (Fig. 4A). We detected high levels of *HTT1a* expression that significantly increased with CAG repeat length. However, repeat sizing of some of the lymphoblastoid lines show cell populations with highly expanded CAG repeats (Fig. 4B, arrows). We detected these expanded populations in all groups; interestingly some control lines expanded into the disease relevant range (Fig. 4B, control), but not systematically in every line.

Next, we wanted to determine, whether most of the cells in the lymphoblastoid lines, which were CD123^+^/CD19^+^ cells (corresponding to a population of dendritic like cells left after immortalisation), were also responsible for the high levels of *HTT1a* expression. To this end, we FACS sorted the dendritic cells and analysed *HTT* exon 2 and *HTT1a* expression levels in comparison to the total population (Fig. 4C). We detected comparable levels of *HTT* exon 2 expression. However, expression levels of *HTT1a* were comparatively lower in the CD123^+^/CD19^+^ cells than in the total population suggesting that another cell type, other than CD123^+^/CD19^+^ cells, was generating the high levels of *HTT1a* (Fig. 4C). CAG repeat sizes in the CD123^+^/CD19^+^ cells were generally the same as in the total population (Fig. 4D). While this was certainly an interesting observation, given the issues around CAG repeat instability in these lines and the generation through EB virus transduction, a careful further evaluation of *HTT1a* expression in different cell types isolated from native blood cells is required in future experiments.

### HTT1a is generated in a CAG repeat-length-dependent manner in PBMCs, but not in skeletal muscle tissue

PBMCs from blood, as well as skeletal muscle, are easily accessible peripheral sample sources. The samples can be collected from living individuals and can be rapidly processed. Therefore, they do not suffer from RNA degradation due to *post mortem* delays. Monitoring *HTT1a* expression in these samples could be of high clinical relevance.

**Fig. 5:**
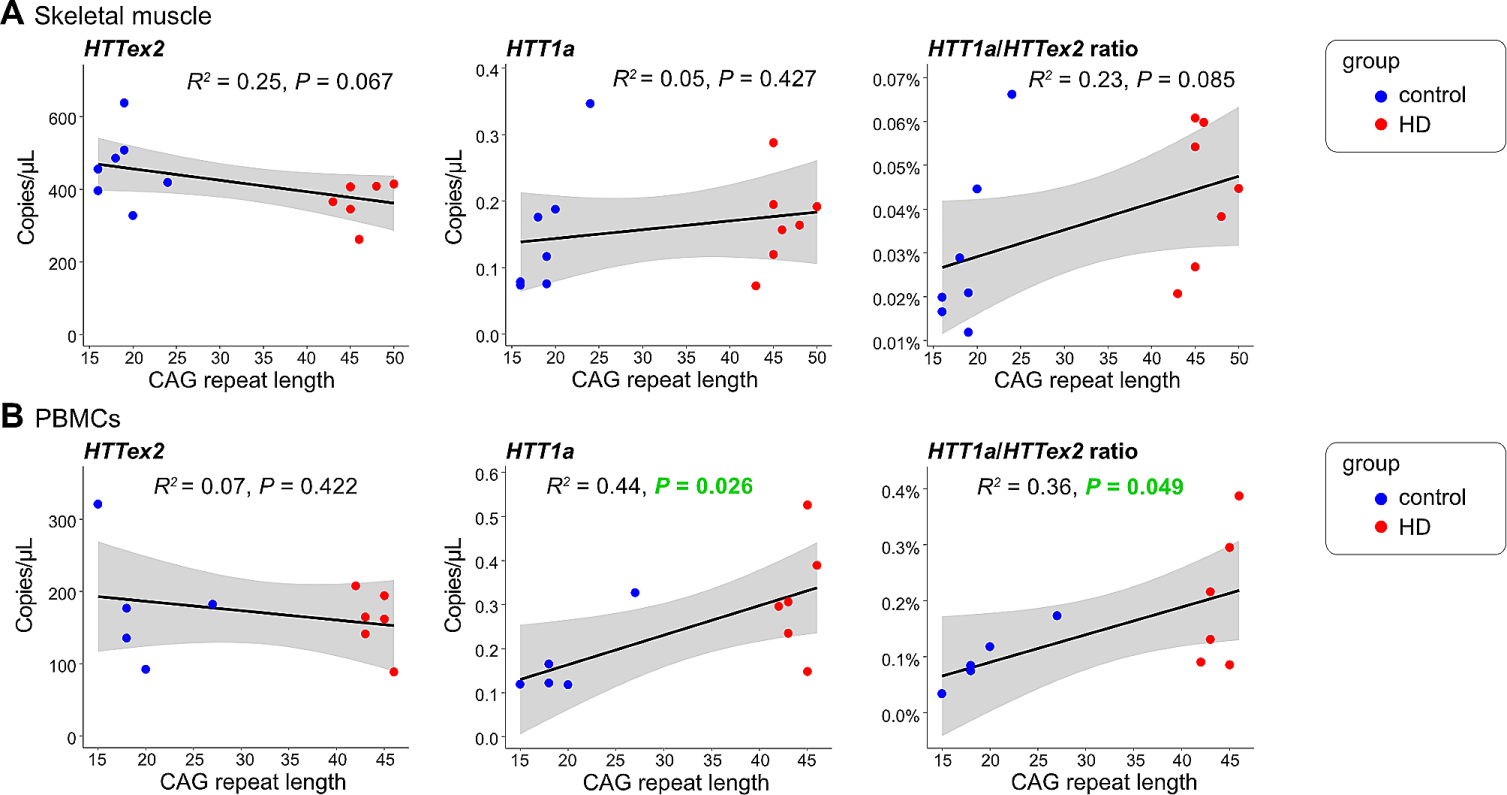
*HTT1a* is generated in a CAG repeat-length-dependent manner in PBMCs, but not in skeletal muscle tissue *HTT1a* and *HTT* exon 2 (*HTTex2*) expression in human skeletal muscle tissue (**A**) or PBMCs (**B**) was analysed from *HTT* mutation carriers (HD) or control individuals (control). The black line represents the linear model of expression level with CAG repeat length. R^2^ (coefficient of determination) and *P*value for the fit of the linear modelling are shown. Grey areas represent the standard error of the regression model.

We analysed skeletal muscle samples and PBMCs from *HTT* mutation carriers in the early manifest stages, as well as age and sex matched control individuals (Fig. 5). *HTT* exon 2 expression in PBMCs was only about half of the levels in skeletal muscles (Fig. 5, *HTTex2*). However, *HTT1a* expression in control individuals was similar in both sample types, indicating either better quality control for splicing in skeletal muscle, or a more severe HD related phenotype in PBMCs as compared to skeletal muscle (Fig. 5, *HTT1a*). Consequently, we detected only slightly elevated expression levels of *HTT1a* in the skeletal muscle samples from *HTT* mutation carriers. In contrast, *HTT1a* levels in PBMCs were significantly higher in *HTT* mutation carriers compared to unaffected individuals and increased with CAG repeat lengths (Fig. 5B).

## Discussion

The mutated CAG repeat tract results in the production of a small RNA, *HTT1a*, coding for the HTT exon1 protein. To study the relationship between CAG repeat length and the amount of *HTT1a* generated, we developed highly sensitive digital PCR assays. These assays allow the absolute quantification of transcript numbers and thus also facilitate the comparison of *HTT1a* levels between tissues, cell types and across different studies. Our assay design uses a unique sequence introduced by the primer used to reverse transcribe the polyA RNAs, thus we do not include unspliced pre-mRNA in our analysis, and hence all signals represent ‘true’ *HTT1a* transcripts, independent of CAG repeat length. Furthermore, the detection of *HTT1a* in samples with CAG lengths in the unaffected range is supported by our current model for the generation of *HTT1a*. In this model, a kinetically controlled splicing disruption and polyadenylation occurs in *HTT* intron 1 due to RNA-polymerase II (PolII) slowing down (Neueder et al. 2018). The main difference would be the likelihood and time window of occurrence, resulting in diminished levels of *HTT1a* for CAG repeat lengths in the unaffected range.

PolII kinetics can also be influenced by epigenetics, i.e. DNA methylation and histone modifications. For example, the binding of factors like CTCF and MeCP2 to unmethylated DNA can slow down the PolII complex and has been found to participate in the alternative splicing of many genes in the brain (Wang et al. 2023). Furthermore, exons and introns can differ in their DNA methylation state and nucleosome occupancy with exons exhibiting higher methylation and nucleosome levels (Lev Maor et al. 2015). Although the ‘epigenetic clock’, which is based on certain DNA methylation marks and used for molecular age assessment, seems to be accelerated in HD samples (Lu et al. 2020), no consistent genome-wide trends for either DNA hypo- or hypermethylation have been observed in HD samples (Lu et al. 2020; Hyeon et al. 2021). It would be most interesting to see if DNA modifications would influence *HTT1a* production, either directly or indirectly through modulation of e.g. PolII kinetics.

In skeletal muscle samples, we found no significant correlation of *HTT1a* expression with CAG repeat length. In two other peripheral sample sources, lymphoblastoid cell lines and PBMCs, we found a statistically significant positive correlation of *HTT1a* expression and CAG repeat length. For these cell types it would also be interesting to analyse if the CAG repeat length dependent generation of *HTT1a* will increase with longer CAG repeat lengths in the juvenile onset range. Both sample types can easily be collected without invasive procedures, do not suffer from *post mortem* delay induced RNA degradation and could thus allow direct monitoring of *HTT1a* levels, for example in the course of a clinical trial aiming to lower *HTT* levels.

Our data of the stability of the CAG repeat in lymphoblastoid cell lines (Fig. 4) suggest that these lines should be used with caution in research concerning somatic CAG repeat instability. We detected a statistically significant positive correlation of *HTT1a* expression levels with CAG repeat lengths. However, our repeat sizing in these lines showed large variations in populations expressing *HTT* alleles with different CAG sizes in the same cell line. Analogous observations have been made by other groups, e.g. the analysis of DNA structural alterations increased with time in culture and was different between DNA regions (Scheinfeldt et al. 2018). The main reason for this observation is most probably the transformation of the original cells with the EB virus inducing genomic instability.

Future analysis of longitudinally matched PBMC samples with a larger spread of CAG repeat lengths and with defined genetic modifier state would show if there is a correlation of *HTT1a* expression with CAG repeat lengths over time and with somatic repeat instability. These data might also help to define the threshold of CAG repeats at which the pathogenic processes become more severe and/or accelerates. Our data, in their current state, are reasonably similar in all tissues to the proposed threshold of about 90–110 CAGs (reviewed in (Donaldson et al. 2021). Larger datasets would also help to explore the linearity or nonlinearity of the *HTT1a*-CAG repeat length correlation. In our paper, we have analysed the data using linear correlations, because we do not have enough data points across a wider range of CAG repeat lengths to be powered for analysis of non-linear correlations. Some previous data suggest that it might indeed be sigmoidal, which needs to be confirmed (Neueder et al. 2018).

Using previously analysed samples and a new set of *post mortem* brain samples, we showed that, in general, the expression levels of *HTT1a* increased with longer CAG tract size in *post mortem* brain tissue. We could only detect elevated levels of *HTT1a* in samples with a CAG repeat length in the juvenile-onset range as compared to control individuals (Fig. 3 and Figure S2). However, as discussed above, the block in splicing might also occur in control individuals. *HTT1a* from HD samples would still encode for an elongated polyglutamine tract and most probably induce a more severe phenotype compared to a HTT exon 1 protein with only a short polyglutamine tract as encoded by *HTT1a* from control individuals. Furthermore, due to phase transitioning by extensive intra- and inter-molecular interactions of the expanded CAG tracts *HTT1a* RNA clusters might not have been fully solubilised during RNA extraction (Ly et al. 2022). Therefore, we might underestimate the amount of *HTT1a* generated, because we only analyse the cytoplasmic, soluble *HTT1a* RNA fraction.

Furthermore, the main driver for elevated expression levels of *HTT1a* in an individual is most probably somatic CAG repeat expansion. Given that extensive somatic CAG expansion seems to be present in only few neuronal subpopulations like striatal medium spiny neurons or the Purkinje neurons of the cerebellum (Matlik et al. 2023), one cannot expect highly elevated levels of *HTT1a* in these tissues on bulk RNA level if the initial repeat length was in the adult-onset range. Our data from juvenile brains, which probably reflect the state of highly expanded neurons in an adult-onset individual, show that the levels of *HTT1a* can reach a high percentage of all *HTT* transcripts (Fig. 3A and B).

In summary, expression of *HTT1a* occurs throughout a wide range of tissues and likely with all CAG lengths. Our data from peripheral sample sources demonstrate that *HTT1a* is indeed generated throughout the body in a CAG repeat-length-dependent manner. Therefore, the levels of *HTT1a* might be a sensitive marker of disease state and/or progression and should be monitored over time, especially in clinical trials targeting *HTT* expression.

## Materials and methods

### Sample collection

Lymphoblastoid cell lines were purchased from the European Huntington Disease Network (EHDN). Collection of other specimen and generation of fibroblast lines has been described previously (Neueder et al. 2017, 2022). Brain tissues were collected at the department of neurology of the University Hospital Ulm (Germany). All samples were snap frozen in liquid nitrogen and stored at -80 ˚C. Information about the samples including measured CAG repeat sizes can be found in Table S1. The ethics committee at Ulm University approved the analyses (ethics applications 265/12 and 252/23). All experimental methods comply with the Helsinki Declaration.

### Cell culture maintenance

Primary human fibroblasts were grown in DMEM (Gibco, #41965-039) with 10% FBS (Pan Biotech, #P30-3031), 100 U/mL Penicillin-Streptomycin (Gibco, #2,441,834), 1x Minimum essential medium non-essential amino acids (Gibco, #11140-035) and 1 mM sodium pyruvate (Gibco, #11360-039) and lymphoblastoid cell lines were cultured in RPMI-1640 (Gibco, #21875-034) with 20% FBS (Pan Biotech, #P30-3031) and 100 U/mL Penicillin-Streptomycin (Gibco, #2,441,834). All cell lines were grown at 37 °C with 5% CO_2_ and passaged when they reached about 90% density to enable stable growth. Adherent cells were detached using 0.05% Trypsin/0.02% EDTA (Pan Biotech, #P10-023100) and lymphoblastoid cell lines were resuspended to disrupt spheroids and generate a single cell suspension. For harvesting, cells were centrifuged at 70 g for 5 min and the pellets were washed with DPBS (Gibco, #14190-094) once. Cells were either directly used for experiments or cell pellets were frozen in liquid nitrogen and stored at -80 °C.

### RNA isolation

RNA from cultured cell lines was isolated using the RNeasy Plus Mini Kit (Qiagen, #74,136) according to manufacturer’s instructions. In brief, the cell pellets were thawed on ice and lysed in 600 µL RLT buffer. The lysates were transferred to gDNA eliminator columns, centrifuged for 30 s at 8000 g and 600 µL of 100% ethanol was added to the flow-through. After mixing 700 µL of sample were transferred to a RNeasy spin column and centrifuged for 15 s at 8000 g. The columns were washed by subsequent addition of 700 µL buffer RW1 and two times 500 µL buffer RPE with centrifugation for 15 s (last step 2 min) at 8000 g. The columns were placed in a fresh collection tube and centrifuged for 1 min at 16,000 g to minimize buffer carryover. RNA was eluted twice using two times 30 µL RNase-free water at 8000 g for 1 min. For RNA extractions of tissues, 30–70 mg of frozen muscle tissue or 70–120 mg of frozen brain tissue were used. Tissue was kept on dry ice and placed in precooled tubes. To improve tissue disruption all pieces of tissue were ground with a liquid nitrogen cooled mortar and pestle. Muscle RNA was isolated using the RNeasy Plus Universal Mini Kit (Qiagen, #1,062,832) and RNA from brain was isolated using the RNeasy Lipid Tissue Mini Kit (Qiagen, #1,023,539) following manufacturer’s instructions. Tissues were dissociated after addition of 900/1000 µL Qiazol using Lysing Matrix D tubes (MP Bio, #6,913,100) and the TissueLyser LT (Qiagen) for 5–10 min at 4 °C and 50 Hz. Visual inspection of tissue disruption was performed, and samples were placed at room temperature (RT) for 5 min. 100 µL of gDNA Eliminator Solution was added to muscle samples and tubes were mixed vigorously. Next, 250 µL (muscle) or 200 µL (brain) of 100% chloroform were added and samples were mixed extensively. The tubes were incubated for 2 min at RT and centrifuged at 12,000 g for 15 min at 4 °C. The aqueous phase was transferred to a fresh tube and mixed with 500 µL of 100% ethanol. Samples were passed through RNeasy spin columns in 700 µL steps by centrifugation for 15 s at 8000 g. Washing was performed with 700 µL of buffer RW1 and two times 500 µL buffer RPE with a last centrifugation step of 2 min at 8000 g. The columns were placed in a fresh collection tube and centrifuged for 1 min at 16,000 g. RNA was eluted into a collection tube using two times 30 µL RNase-free water at 8000 g for 1 min (muscle) or only one time 30 µL RNase-free water at 8000 g for 1 min (brain). PBMC RNA was isolated with the AllPrep DNA/RNA/Protein Mini Kit (Qiagen, #80,004) according to manufacturer’s instructions. Pellets were thawed on ice, loosened by thorough flicking and resuspend in 600 µL RLT buffer containing 0.14 M β-mercaptoethanol. Samples were homogenized by passing the lysate six times through a 0.8 mm needle using a 1 mL syringe. Samples were transferred to ALLPrep DNA spin columns and centrifuged for 30 s at 8000 g. DNA columns were stored at 4 °C and the flow-through was mixed with 400 µL of 100% ethanol. The samples were transferred to RNeasy spin columns and centrifuged for 15 s at 8000 g. 700 µL RW1 and 2 500 µL RPE were used for washing and the optional buffer removing centrifugation was done at 16,000 g for 1 min. RNA was eluted twice with 30 µL RNase-free water at 8000 for 1 min. All RNA concentrations were determined using the Qubit™ RNA Broad Range Assay Kit (Invitrogen, #Q10211) and a Qubit™ 4.0 Fluorometer (Invitrogen, #Q33226) with 1 µL of undiluted or 1 µL of 1:5 in water diluted sample. In cases where RNA concentrations were lower than needed RNA was precipitated. To this end, the volume of RNA sample was topped up to 100 µL and NaCl was added to a final concentration of 0.3 M. 300 µL of 100% ethanol was added, samples were vortexed for 5 s and incubated at -20 °C overnight. RNA was pelleted by centrifugation at 4 °C for 30 min at 16,000 g. The supernatant was discarded, and the pellets were briefly dried. The RNA was resuspended in RNase-free water and concentrations were measured as described above.

### cDNA synthesis

cDNA was reverse transcribed from 2 µg of RNA using the SuperScript IV Reverse Transcriptase (Invitrogen, #18,090,050) according to manufacturer’s protocol. Each reaction was set up in 20 µL total reaction volume with 0.5 µM UAPdT18 primer, 0.5 mM dNTPs, 1x SSIV Buffer, 5 mM DTT, 2.0 U/µL RNaseOUT RNase Inhibitor (Invitrogen, #10,777,019) and 10 U/µL SuperScript IV Reverse Transcriptase. UAP primer was annealed to the RNA at 65 °C for 5 min; the samples were snap cooled on a pre-chilled metal block for 1 min and the RT mix was added. Reverse transcription was performed at 50 °C for 10 min and inactivation was carried out at 80 °C for 10 min immediately afterwards. cDNA samples were diluted 1:5 in RNase-free water for digital PCR analysis.

### FACS analysis

FACS (fluorescence-activated cell sorting) was performed on lymphoblastoid cell lines to retrieve a homogenous subpopulation of CD123^+^ CD19^+^ cells. Cells were counted using a Neubauer Chamber, washed with DPBS and incubated in blocking buffer (DPBS with 5% (v/v) FBS) for 15 min before the cells were diluted to a concentration of 1 × 10^7^ cells/mL. Subsequently, 2 × 10^7^ cells were labelled with 17.5 µL α-CD123-PE-Cy^TM^7 (BD Bioscience, #560,826) and 80 µL α-CD19-FITC (BD Bioscience, #555,412) antibodies for 45 min at 4 °C on an end-over-end rotator in the dark. Cells were washed with FACS buffer (DPBS with 0.1% (v/v) FBS) once and passed through a 40 μm nylon cell strainer (Corning Inc., #431,750) into FACS tubes. Unstained and single-colour control samples were prepared likewise without or single addition of antibodies respectively. Cells were sorted with the FACSMelody™ Cell Sorter (BD Bioscience) using the FACSChorus™ Software (BD Bioscience). Gating for live cells (P1) was done using FSC-A/SSC-A and single cells of P1 were identified with SSC-W/SSC-H (P2) and FSC-W/FSC-H (P3). Cells of P3 double positive for CD123 and CD19 were then sorted. The sorted samples were further analysed in parallel with the bulk sample pellet using dPCR. FACS evaluation analysis was performed using FlowJo™ Software (BD Biosciences).

### CAG repeat sizing

CAG repeat sizing PCR was performed on cDNA from lymphoblastoid cell lines in 10 µL reactions containing 5 µL Q5® High-Fidelity 2X Master Mix (New England Biolabs, #M0492S), 0.5 µM forward primer (5’ - [FAM] - ATG AAG GCC TTC GAG TCC CTC AAG TCC TTC), 0.5 µM reverse primer (5’ - GGC GGC TGA GGA AGC TGA GGA), 3 µL ddH2O and 1 µL sample. Thermal cycling was as follows: 98 °C for 30 s; 45 cycles of 98 °C for 10 s and 72 °C for 90 s; final extension at 72 °C for 2 min. 1 µL of PCR product was mixed with 9.5 µL formamide (Sigma-Aldrich, #47,671) and 0.5 µL internal standard WEN ILS 500 (Promega, #DG5001). Samples were boiled at 99 °C for 2 min and then placed on ice. Gel capillary electrophoresis was performed with an ABI 3030 standard capillary for 8500 s using the ABI Hitachi 3130 DNA Genetic Analyzer Sequencer (Applied Biosystems, #3130XLR). Analysis of CAG repeat lengths was performed with GeneMapper™ (Applied Biosystems, #4,475,073). The size of the CAG repeat indicated by the highest peak was calculated according to the following equation: (peak position [bp] − 78 bp) / 3.

### Digital PCR

Digital PCR (dPCR) was performed on cDNA samples from tissue and cell lines using the QuantStudio™ 3D Digital PCR System. Primers and probes (Table 1) were combined into an assay mix with a final concentration of 3.75 pmol/µL per primer and 2.5 pmol/µL per probe. A master mix containing 7.25 µL of 2x QuantStudio™ 3D Digital PCR Master Mix V2 (Applied Biosystems, #A26358) and 0.75 µL assay mix was added to 6.5 µL of cDNA sample per chip (Applied Biosystems, #A26316). Samples were run as duplicates on two chips for all main data except data in Fig. 1C. Thermal cycling was performed on a ProFlex™ 2 x flat PCR System with cycling conditions as follows: 1 step of 96.0 °C for 10 min 50 steps of 55.0 °C for 2 min and 98.0 °C for 30 s. Final extension at 55.0 °C for 2 min followed by 10 °C on hold. The chips were allowed to reach room temperature in the dark and the fluorescent signals were then measured with the QuantStudio™ 3D Digital PCR System (Applied Biosystems, #4,481,097).

### Digital PCR evaluation

Data was analysed using the QuantStudio™ 3D AnalysisSuite Software (Applied Biosystems, version 3.1.6-PCR-build18). Rare mutation detection was enabled, the quality threshold for each well was set to 0.5 and the Poisson Plus algorithm (version 04.04.2010) with a confidence level of 95% and a desired precision of 5% was used for quantification. The threshold for rare calls was adjusted in the 3D AnalysisSuite software for each experiment while keeping it as low as possible. Thresholding was chosen to fit the visual separation of populations in positive control samples and other samples with clear population separation. Additionally, it was aimed for the no-template controls and wild-type samples to show minimal positive signals. All other statistical analyses were conducted in R v4.3.0 (Team 2022).

### Electronic supplementary material

Below is the link to the electronic supplementary material.

Supplementary Material 1

## Data Availability

Further details about the analysis pipeline, as well as scripts and code are available upon reasonable request from A.N. (andreas.neueder@uni-ulm.de).
