# Correction: Huntingtin *HTT1a* is generated in a CAG repeat-length-dependent manner in human tissues

**DOI:** 10.1186/s10020-024-00810-1

**Published:** 2024-04-10

**Authors:** Franziska Hoschek, Julia Natan, Maximilian Wagner, Kirupa Sathasivam, Alshaimaa Abdelmoez, Björn von Einem, Gillian P. Bates, G. Bernhard Landwehrmeyer, Andreas Neueder

**Affiliations:** 1 Department of Neurology, University Hospital Ulm, 89081 Ulm, Germany; 2 Huntington’s Disease Centre, Department of Neurodegenerative Disease, Queen Square Institute of Neurology, University College London, WC1N 3BG London, UK; 3 Department of Pharmaceutical Organic Chemistry, Faculty of Pharmacy, Assiut University, Assiut, Egypt

Molecular Medicine (2024) 30:36 https://doi.org/10.1186/s10020-024-00801-2

Following publication of the original article, it came to the journal's attention that the captions of the figures were incomplete. The figure legends have since been corrected in the published article. The journal and the authors thank you for reading this erratum.

