# Supplementary material for: Huntingtin HTT1a is generated in a CAG repeat-length-dependent manner in human tissues
Source: Mol Med. 2024 Mar 8;30:36. doi: 10.1186/s10020-024-00801-2 (PMC10924374; doi:10.1186/s10020-024-00801-2)
Supplement: Supplementary file 1 — Supplementary Material 1 [file 10020_2024_801_MOESM1_ESM.pdf]

Huntingtin *HTT1a* is generated in a CAG repeat-length-dependent manner in human tissues

Franziska Hoschek[1], Julia Natan[1], Maximilian Wagner[1], Kirupa Sathasivam[2], Alshaimaa Abdelmoez[1,3], Björn von Einem[1], Gillian P. Bates[2], G. Bernhard Landwehrmeyer[1], Andreas Neueder[1,*]

[1]Department of Neurology, University Hospital Ulm, 89081 Ulm, Germany

[2]Huntington's Disease Centre, Department of Neurodegenerative Disease, Queen Square Institute of Neurology, University College London, London WC1N 3BG, United Kingdom

[3]Department of Pharmaceutical Organic Chemistry, Faculty of Pharmacy, Assiut University, Assiut, Egypt

***Correspondence**

Andreas Neueder
Department of Neurology, University Hospital Ulm, 89081 Ulm, Germany
P: +49 731 500 63117
E: andreas.neueder@uni-ulm.de
ORCID: 0000-0002-2389-5236

Supporting Information

Supplementary Figures and Figure legends

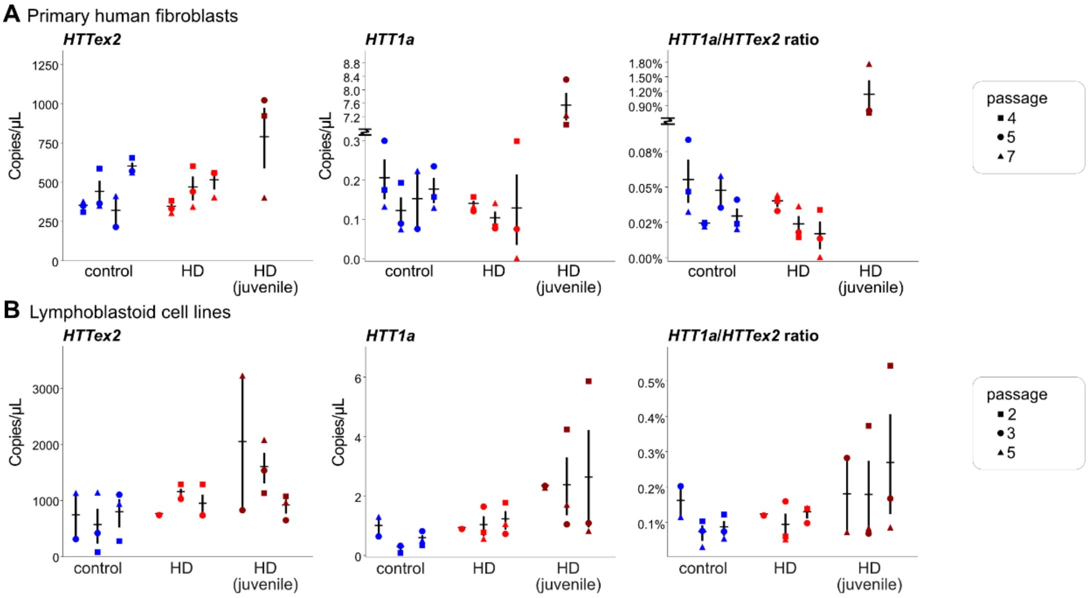

Figure S1. Time in culture has no major influence on *HTT1a* or *HTT* exon 2 expression in primary human fibroblasts and lymphoblastoid cell lines

HTT1a and *HTT* exon 2 (*HTTex2*) expression in primary human fibroblast (**A**) and lymphoblastoid lines (**B**) was analysed in lines with a CAG repeat in the control (control), adult-onset (HD) or juvenile-onset range (HD juvenile). Each data point stack corresponds to one line with three passages as individual data points (squares, circles, triangles). CAG sizes and passage number were used as main effects in two-way ANOVA analyses and no significant differences were found.

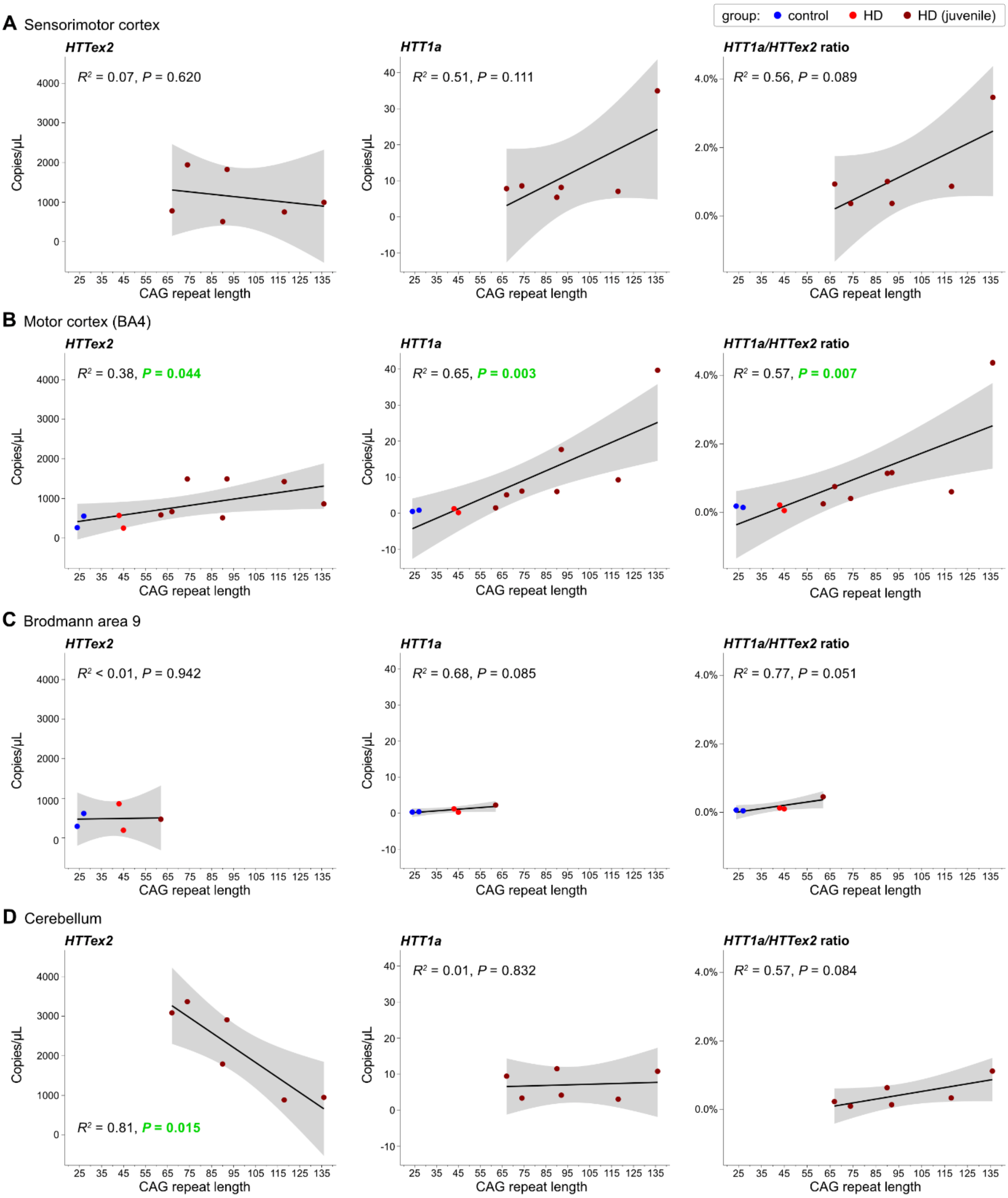

Figure S2. *HTT1a* is generated in a CAG repeat-length-dependent manner in *post mortem* motor cortex

HTT1a and *HTT* exon 2 (*HTTex2*) expression levels in sensorimotor cortex (**A**), motor cortex (BA4) (**B**), BA9 cortex (**C**) and cerebellum (**D**) were analysed from *HTT* mutation carriers with adult-onset CAG repeat lengths (HD) or juvenile-onset CAG repeat lengths (HD juvenile) and control individuals (control), respectively. The black line represents the linear model of expression level with CAG repeat length. R^2 (coefficient of determination) and *P* value for the fit of the linear modelling are shown. Grey areas represent the standard error of the regression model.

Supplementary Tables

Table S1. Sample Information

Tissue	Sample ID	HD status	CAG repeat length
Post mortem brain[1]	FR_13	control	24
	FR_14	control	27
	UB_03	adult	45
	UB_11	adult	43
	UB_10	juvenile	62
	B5817	juvenile	67
	B5345	juvenile	74
	B5928	juvenile	90
	B4522	juvenile	92
	B4383	juvenile	118
	B5504	juvenile	136
Primary human fibroblasts[2]	MTMHD_53	control	16
	MTMHD_13	control	16
	MTMHD_23	control	16
	GM02155	control	17
	MTMHD_07	control	18
	MTMHD_06	adult	44
	MTMHD_62	adult	44
	MTMHD_16	adult	44
	GM03621	juvenile	61
	GM04737	juvenile	64
	GM04723	juvenile	70
	GM04281	juvenile	72
	GM05539	juvenile	98
	GM09197	juvenile	177
PBMCs[3]	MTMHD_45	control	27
	MTMHD_26	control	20
	MTMHD_40	control	18
	MTMHD_68	control	15
	MTMHD_56	control	18
	MTMHD_42	adult	45
	MTMHD_41	adult	43
	MTMHD_05	adult	43
	MTMHD_67	adult	46
	MTMHD_63	adult	42
	MTMHD_08	adult	45

Tissue	Sample ID	HD status	CAG repeat length
Skeletal muscle[4]	MTMHD_35	control	19
	MTMHD_21	control	18
	MTMHD_26	control	20
	MTMHD_23	control	16
	MTMHD_24	control	19
	MTMHD_53	control	16
	MTMHD_50	control	24
	MTMHD_20	adult	45
	MTMHD_27	adult	48
	MTMHD_38	adult	45
	MTMHD_42	adult	45
	MTMHD_41	adult	43
	MTMHD_65	adult	50
	MTMHD_52	adult	46
Lymphoblastoid cells[5]	EN-110915-20195	control	17
	EN-271015-00367	control	18
	EN-241215-05520	control	23
	EN-120915-09734	adult	42
	EN-110915-03607	adult	41
	EN-120915-16051	adult	43
	EN-120915-37418	juvenile	67
	EN-110915-21894	juvenile	62
	EN-120915-20923	juvenile	60
	EN-120915-43070	juvenile	60
	HR-300915-21905	juvenile	66

CAG sizes for *post mortem* brains[1] (BXXXX) and primary fibroblasts[2] (GMOXXXX) samples have been partly published in [1]. CAG sizes for PBMCs[3] and skeletal muscle[4] samples (MTMHD_XX) have been published in [2]. CAG sizes of lymphoblastoid cells[5] (EN-X…) were and all other repeat sizes were determined by the authors. Consistency between the published and newly defined CAG sizes was ensured by reanalysis of several samples.

Supplementary references

1. Neueder, A., C. Landles, R. Ghosh, D. Howland, R.H. Myers, R.L.M. Faull, S.J. Tabrizi, and G.P. Bates, *The pathogenic exon 1 HTT protein is produced by incomplete splicing in Huntington's disease patients.* Sci Rep, 2017. 7(1): p. 1307. DOI: 10.1038/s41598-017-01510-z
2. Neueder, A., K. Kojer, T. Hering, D.J. Lavery, J. Chen, N. Birth, J. Hallitsch, S. Trautmann, J. Parker, M. Flower, et al., *Abnormal molecular signatures of inflammation, energy metabolism, and vesicle biology in human Huntington disease peripheral tissues.* Genome Biol, 2022. 23(1): p. 189. DOI: 10.1186/s13059-022-02752-5
